# Site-specific identification of heparan and chondroitin sulfate glycosaminoglycans in hybrid proteoglycans

**DOI:** 10.1038/srep34537

**Published:** 2016-10-03

**Authors:** Fredrik Noborn, Alejandro Gomez Toledo, Anders Green, Waqas Nasir, Carina Sihlbom, Jonas Nilsson, Göran Larson

**Affiliations:** 1 Department of Clinical Chemistry and Transfusion Medicine, Institute of Biomedicine, University of Gothenburg, Sahlgrenska University Hospital, SE-413 45 Gothenburg, Sweden; 2 Proteomics Core Facility, Sahlgrenska Academy, University of Gothenburg, Box 413, SE-405 30, Sweden

## Abstract

Heparan sulfate (HS) and chondroitin sulfate (CS) are complex polysaccharides that regulate important biological pathways in virtually all metazoan organisms. The polysaccharides often display opposite effects on cell functions with HS and CS structural motifs presenting unique binding sites for specific ligands. Still, the mechanisms by which glycan biosynthesis generates complex HS and CS polysaccharides required for the regulation of mammalian physiology remain elusive. Here we present a glycoproteomic approach that identifies and differentiates between HS and CS attachment sites and provides identity to the core proteins. Glycopeptides were prepared from perlecan, a complex proteoglycan known to be substituted with both HS and CS chains, further digested with heparinase or chondroitinase ABC to reduce the HS and CS chain lengths respectively, and thereafter analyzed by nLC-MS/MS. This protocol enabled the identification of three consensus HS sites and one hybrid site, carrying either a HS or a CS chain. Inspection of the amino acid sequence at the hybrid attachment locus indicates that certain peptide motifs may encode for the chain type selection process. This analytical approach will become useful when addressing fundamental questions in basic biology specifically in elucidating the functional roles of site-specific glycosylations of proteoglycans.

Polysaccharides constitute a wide range of structures that are essential components for all living organisms. In contrast to the template-driven synthesis of nucleic acids and proteins, the syntheses of polysaccharides depend on numerous of enzymes working sequentially to yield glycan structures of significant heterogeneity^1^,^2^. While some polysaccharides, such as cellulose and starch, have relatively simple structures, others, such as glycosaminoglycans (GAGs), that regulate key processes in development and homeostasis, display considerable structural complexity^3^,^4^. Heparan sulfate (HS) and chondroitin sulfate (CS) constitute two important subfamilies of GAGs and these polysaccharides interact with a multitude of protein ligands, which mediate events such as angiogenesis, neurogenesis and inflammation^5^. Distinct HS and CS structural motifs have been identified as binding sites for various protein ligands and determining the relevance of these GAG-protein interactions is a major research area^6^,^7^,^8^,^9^. However, the specific mechanisms by which glycan biosynthesis generates complex HS and CS polysaccharides, required for the regulation of mammalian physiology, remain to be elucidated.

The HS and CS polysaccharides are covalently attached to core proteins of various heparan sulfate (HSPGs) and chondroitin sulfate proteoglycans (CSPGs). While certain core proteins consistently carry HS, others carry CS and still others are hybrid proteoglycans carrying both HS and CS chains. The GAG biosynthesis is initiated with the enzymatic transfer of a beta-linked xylose (Xyl) to specific Ser residues of the core protein sequence^10^. The synthesis continues with the enzymatic addition of two galactoses (Gal) and one glucuronic acid (GlcA) residue, finalizing the formation of a characteristic tetrasaccharide linkage region (GlcAβ3Galβ3Galβ4XylβSer). In the next step the enzymatic addition of an N-acetylglucosamine (GlcNAcα4) primes the synthesis to HS chains, whereas the addition of an N-acetylgalactosamine (GalNAcβ4) leads to the synthesis of CS chains. The elongation of HS chains continues through the addition of repeating units of GlcA and GlcNAc residues, whereas addition of repeating units of GlcA and GalNAc forms CS chains. The CS and HS chains are thereafter extensively modified by class-specific epimerases and sulfotransferases^11^,^12^,^13^. The uniquely glycosylated Ser residues of the core proteins are usually flanked by a glycine residue (-SG-) and certain features have been identified that influence the selection of HS synthesis vs CS synthesis. The presence of repetitive SG motifs seems to prime for HS synthesis, whereas a single SG motif in combination with a cluster of acidic residues in close proximity seems to prime for CS^10^. However, due to the structural complexity of proteoglycans and the limitations of the present analytical techniques, the influence of the peptide sequence for GAG-glycosylation has not yet been fully explored.

Novel strategies using tandem mass spectrometry (MS/MS) have recently been developed that provide site-specific structural information of N- and O-glycans attached to various tryptic peptides in a bottom-up strategy to characterize glycoproteins. Such strategies are referred to as ‘glycoproteomics’ and are typically based on an initial and specific enrichment step of glycopeptides and a subsequent analysis with nano-liquid chromatography-tandem mass spectrometry (nLC-MS/MS)^14^,^15^. Glycoproteomics has resulted in the identification of hundreds of novel N- and O- glycosylation sites and recent methods have swiftly become essential tools in cell biology and biomedical research^16^,^17^. More recently, we introduced a similar bottom-up approach to characterize CSPGs that enabled site-specific identification of novel and established core proteins and the mapping of unforeseen structural complexity of the linkage region of bikunin^18^,^19^. With this methodology at hand we now wanted to further develop this concept of characterizing hybrid proteoglycans as to both CS and HS chain structures and their respective attachment sites. Thus, a similar approach for characterization of HSPGs had to be developed and as the first proteoglycan for study we chose perlecan and, for reasons of availability, a commercial sample derived from Engelbreth-Holm-Swarm mouse sarcoma. Perlecan (also known as basement membrane specific heparan sulfate proteoglycan, coded for by the gene *HSPG2*) has been the focus of previous structural studies and is perceived as relatively well characterized^20^,^21^. The proteoglycan represents one of the largest extracellular matrix proteins identified (470 kDa) and has a large number of post-translational modifications, which includes three substitutions with HS chains at the N-terminal end as well as several O- and N-linked glycosylations^22^. Moreover, perlecan may also be substituted with CS chains and we recently identified a CS-attachment site at the C-terminal end (Fig. 1a), thus making perlecan a suitable model for addressing the issue of site-specific characterization of hybrid proteoglycans^18^,^23^.

The mouse sarcoma perlecan sample was digested with trypsin and enriched for glycopeptides using strong anion exchange (SAX) chromatography. The resulting fractions were digested with a mixture of heparinases which generate the release of internal disaccharide residues and a residual glycan structure still attached to the peptide. This residual glycan structure is expected to be composed of the linkage region, extended with varying numbers of GlcA-GlcNAc disaccharides where the terminal disaccharide is dehydrated on the hexuronic acid to form delta hexuronic acid (ΔHexA). The generated glycopeptides were analyzed with nLC-MS/MS in positive mode, which allowed for the combined sequencing of the residual glycan structure and of the core peptide. In addition to perlecan, the method enabled site-specific identification of three additional HSPGs also present in the perlecan preparation. The combined use of heparinase and chondroitinase ABC further enabled the identification and differentiation of consensus HS-sites and a hybrid GAG-site of perlecan, carrying either a HS or a CS chain. Inspection of the amino acid sequence at the attachment loci indicated that certain peptide motifs may encode for the chain type selection process. In principle, this analytical approach may be generally used to study the attachment sites and glycan structures of native hybrid proteoglycans, the influence of altered peptide sequences in selecting HS- versus CS-biosynthesis in various cell systems, as well as elucidating the functional roles of site-specific glycosylations of various proteoglycans.

## Results

### Analysis of perlecan GAG-composition with SDS-PAGE

**Figure 1:**
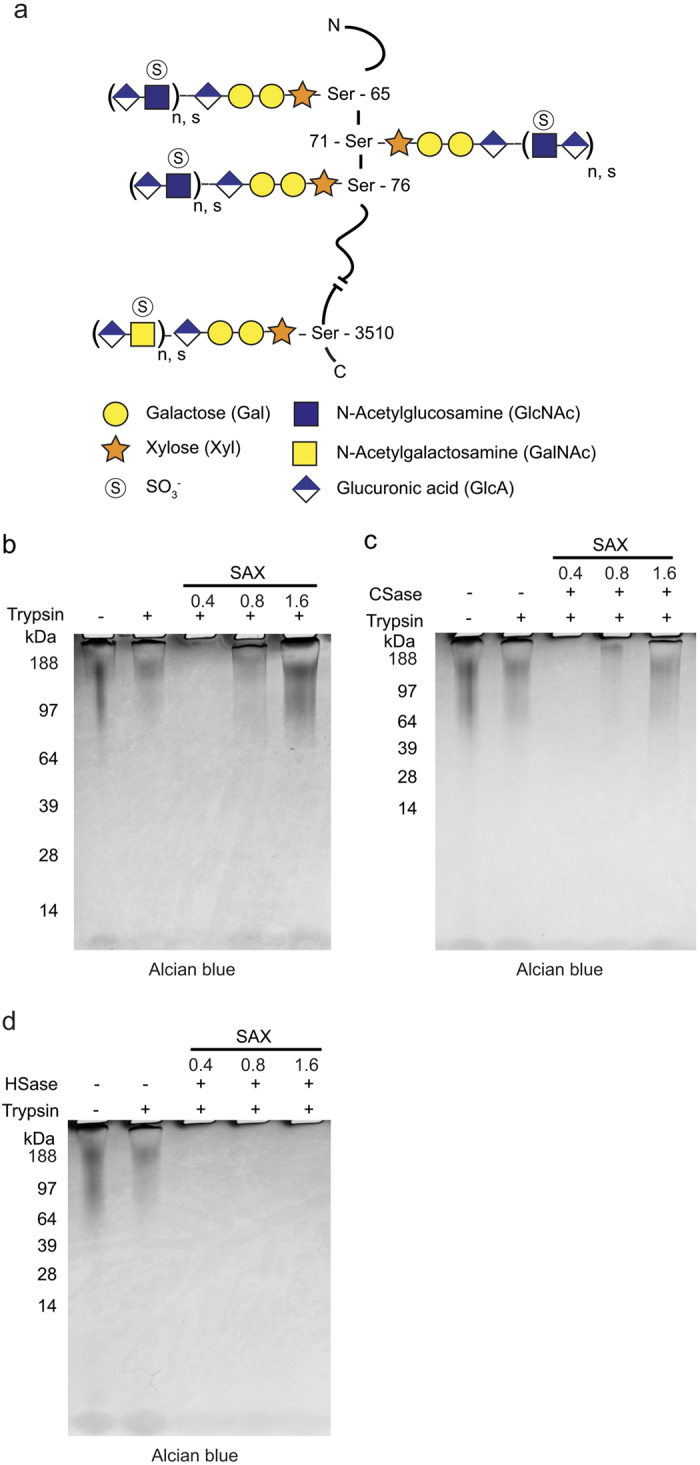
Analysis of perlecan GAG-composition with SDS-PAGE. (**a**) Schematic illustration of perlecan. The proteoglycan may be substituted with GAGs at various positions, including three HS-chains at the N-terminal end as well as a CS-chain at the C-terminal end^18^. The number of disaccharides (n) and the degree of sulfation may vary at each GAG-position. (**b**) Trypsin-digested mouse perlecan was applied onto a SAX-column and enriched for GAG-glycopeptides (see Methods). The GAG-glycopeptides were eluted from the column with increasing salt concentrations (0.4 M, 0. 8 M and 1.6 M NaCl) and analyzed with SDS-PAGE / Alcian blue. Perlecan incubated with and without trypsin was loaded as control. (**c**,**d**) An identical set of SAX-enriched GAG-glycopeptides were treated with either chondroitinase ABC (CSase) (**c**) or heparinase (HSase) (**d**) prior to the SDS-PAGE / Alcian blue analysis.

The relative proportion of HS and CS chains on perlecan was assessed using SDS-PAGE. To obtain defined GAG-glycopeptides for structural analysis, a perlecan sample was incubated with trypsin and passed over a SAX-column, equilibrated with a low-salt buffer (0.2 M NaCl). The positively charged matrix retains anionic polysaccharides and their attached peptides, whereas neutral and positively charged peptides flow through. After a washing step, the bound GAG-glycopeptides were eluted stepwise with three buffers of increasing sodium chloride concentration (0.4 M NaCl, 0.8 M NaCl and 1.6 M NaCl). The three fractions were desalted and analyzed with SDS-PAGE followed by Alcian blue staining to visualize the presence of acidic GAG-chains. As a control, perlecan samples incubated with and without trypsin were loaded onto the gel without any prior SAX-chromatography. The SAX-enriched GAG-glycopeptides migrated as a continuous band on the top section of the gel and were mainly recovered in the 0.8 M and 1.6 M NaCl fractions (Fig. 1b). To assess the relative proportions of CS versus HS, the enriched fractions were treated with either chondroitinase ABC or heparinase prior to the SDS-PAGE and Alcian blue staining. Digestion with chondroitinase resulted in slightly less Alcian blue staining compared with the non-treated fractions, indicating the presence of small amounts of CS chains (Fig. 1c). After heparinase digestion no staining was visible, indicating that the vast majority of the polysaccharides are of HS type (Fig. 1d). Taken together, this confirms that HS is indeed the major GAG type in the sample and inspection of the PAGE profiles indicates that CS constitutes less than 10 % of the total GAG-chains. Nevertheless, these findings support the concept that perlecan may appear as a hybrid proteoglycan.

### Analysis of heparan sulfate glycopeptides

**Figure 2:**
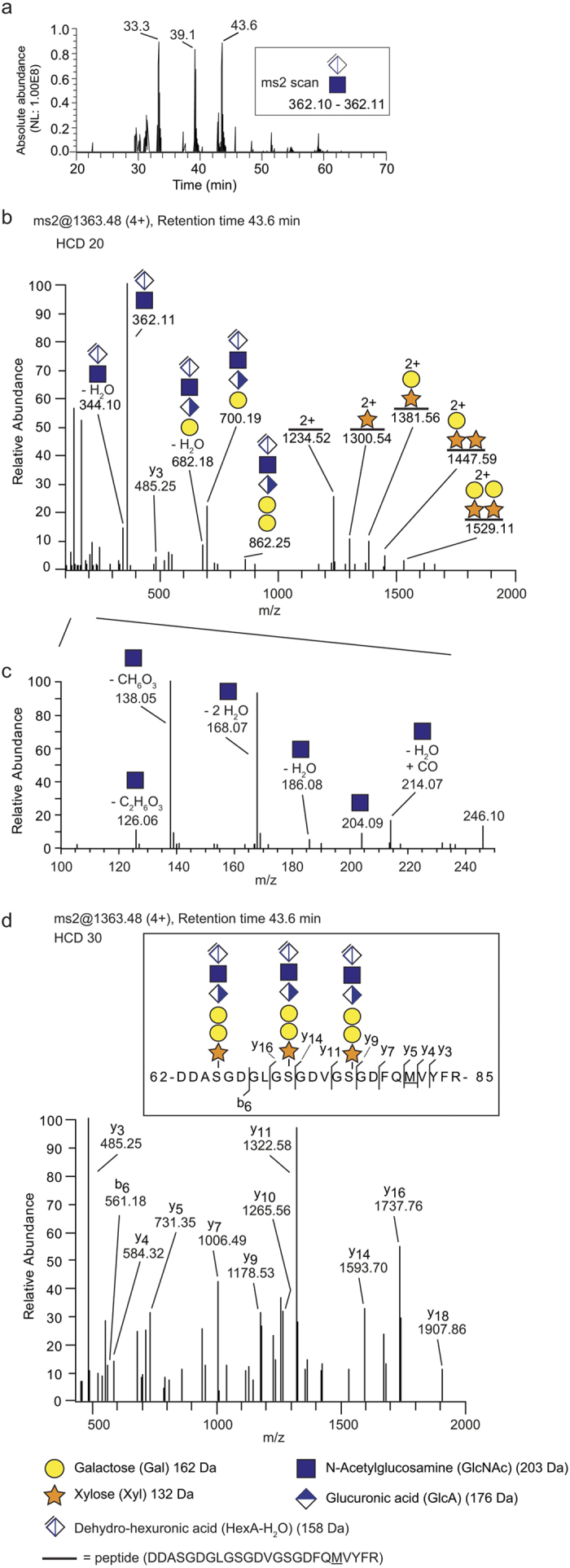
Site-specific analysis of heparan sulfate linkage region glycopeptides. (**a**–**d**) Trypsin-digested perlecan was applied onto a SAX-column and enriched for GAG-glycopeptides. The resulting preparation was digested with heparinase and analyzed with nLC-MS/MS. (**a**) An extracted-ion current chromatogram of MS2-spectra which have been filtered for the presence of the *m/z* 362.11 diagnostic ion, representing the dehydrated disaccharide ion [ΔHexAGlcNAc]^+^, revealed several 362-related peaks eluting at various positions (**b**) An HS-glycopeptide (1363.48; 4+) was identified at 43.6 min and the MS2-spectrum (NCE 20%) showed characteristic glycosidic fragments. (**c**) Enlarged view of the low mass range (*m/z* 100–250) enabled the identification of several GlcNAc-derived oxonium ions. (**d**) MS2-fragmentation at higher energy (NCE 30%) provided several y-ions and one b-ion. The HS-glycopeptide is derived from the N-terminal domain of perlecan (**d**, insert). All ions are [M + zH]^+^ and their charges are shown as superscript when ***z ***> 1.

We then tested whether nLC-MS/MS analysis could be used for site-specific analysis of the HS-glycopeptides. After SAX-chromatography the 1.6 M fraction was digested with heparinase and analyzed with positive mode nLC-MS/MS. To increase the likelihood of generating glycan-specific fragments, the glycopeptides were fragmented at normalized collision energy (NCE) of 20%, as this relatively low energy level generates abundant glycosidic fragmentation^24^,^25^. Inspection of the resulting spectra revealed that the MS2-fragmentation generated intense oxonium ions at *m***/***z* 362.11, corresponding to a terminal dehydrated disaccharide structure [ΔHexAGlcNAc]^+^. The MS2-spectra were therefore filtered for the presence of *m/z* 362.1 (*m/z* range 362.10–362.11) and several 362.1-peaks were indeed identified at various elution times (Fig. 2a). Examination of the peak at 43.6 min displayed a spectrum with abundant glycosidic fragmentation (Fig. 2b,c). In addition to the ion at *m/z* 362.11, other fragment ions were also identified, including a tetrasaccharide- ([ΔHexAGlcNAcGlcAGal]^+^, *m/z* 700.19) and a pentasaccharide ([ΔHexAGlcNAcGlcAGalGal]^+^, *m/z* 862.25) (Fig. 2b). This indicates that the heparinase digestion generated a hexasaccharide structure, composed of the linkage region tetrasaccharide extended with a GlcA-GlcNAc disaccharide, dehydrated on the terminal hexuronic acid (ΔHexA-GlcNAc). The monoisotopic mass of the precursor ion (1362.72; 4+) equated to the mass of a peptide with a DDASGDGLGSGDVGSGDFQMVYFR sequence, derived from the N-terminal domain (amino acids 62–85) of mouse perlecan, encompassing the three previously described HS-attachment sites. The peptide was found to be modified with three hexasaccharide structures and one methionine oxidation. The measured mass (5446.8697 Da) deviated +0.66 ppm from the theoretical value. Detailed inspection also revealed several xylose (132 Da) (e.g. peptide + xylose, *m/z* 1300.54; 2+) and galactose shifts (162 Da) (e.g. peptide + xylose + galactose, *m/z* 1381.56; 2+), further demonstrating the GAG-nature of the structure (Fig. 2b). Notably, fragmentation of glycoproteins often generates various types of glycosidic fragments. Here, the largest glycosidic fragment consisted of the peptide with two of the three serines substituted with one xylose and one galactose respectively (*m/z* 1529.11; 2+). Further, detailed inspection in the low mass range (*m/z* 100–250) enabled the identification of several diagnostic HexNAc-derived oxonium ions. Such ions were the result of H_2_O losses from the *m/z* 204.09 [HexNAc]^+^ ions into *m***/***z* 168.07 and *m***/***z* 186.08, and further decompositions into *m***/***z* 126.06 and *m***/***z* 138.05 (Fig. 2c). Furthermore, an increase of the NCE level to 30% was used to generate abundant peptide fragmentation that enabled the identification of several diagnostic b- and y-ions of the peptide (*m***/***z* 370–2000) (Fig. 2d). The *m/z* range was here started at a higher value to exclude the prominent ion at *m/z* 362.11 that otherwise possibly would suppress the intensities of the weaker b- and y- ions. The identified HS-glycopeptide structure is shown in Fig. 2d, insert.

**Figure 3:**
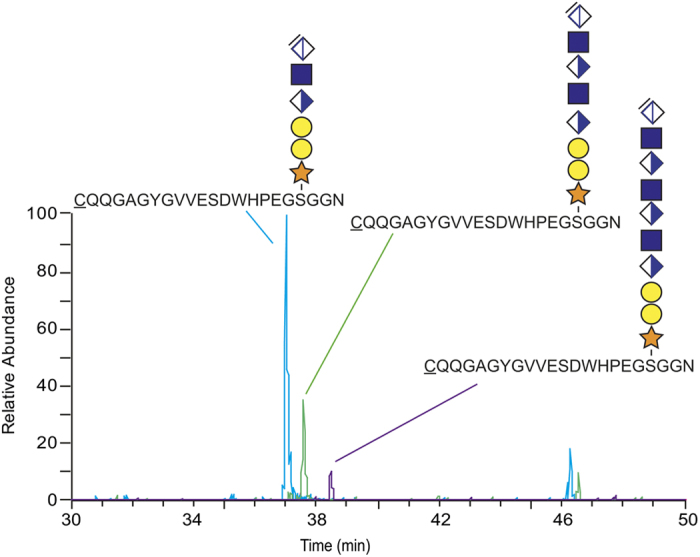
Identification of a HS-site in the C-terminal end of perlecan. Extracted-ion current chromatograms of a heparinase-digested perlecan sample show residual HS-saccharide glycopeptides originating from to the C-terminal perlecan site. The HS-structures shown are hexasaccharides (*m/z* 1095.41; 3+), octasaccharides (*m/z* 1221.78; 3+) and decasaccharides (*m/z* 1348.48; 3+). Notably, the small peaks eluting at ~46 min relates to NH_3_-adducts variants of the native structures.

An automated Mascot search algorithm was constructed to identify if other HS-glycopeptides were present in the sample preparation. This proteomic analysis was allowed to include the hexasaccharide structures ΔHexAGlcNAcGlcAGalGalXyl-O- (993.2808 Da) and was used on the MS-data of the 0.4 M, 0.8 M and 1.6 M fractions. The search algorithm enabled the identification of other extracellular matrix HSPGs, including mouse collagen XVIII and agrin, found as three and one glycopeptide variants, respectively (Supplementary Fig. S1). Surprisingly, a HS-site was identified in the C-terminal end of mouse perlecan (CQQGAGYGVVESDWHPEGSGGN) at the same location that we previously identified a CS-site in human tissue fluids^18^. The identified peptide, corresponding to amino acids 3492–3513, was found substituted with a HS-hexasaccharide (*m/z* 1095.41; 3+), -octasaccharide (*m/z* 1221.78; 3+) or -decasaccharide (*m/z* 1348.16; 3+) (Fig. 3, Supplementary Fig. S2). Tryptic variants of this glycopeptide are indicated in Fig. 2a at 33.3 min and at 39.1 min.

Furthermore, detailed evaluation of additional heparinase-generated glycopeptides revealed that the peptides could also be substituted with tetrasaccharides, thus demonstrating that the enzyme digestion may generate additional length variations. The tetrasaccharides were composed of the linkage region with a dehydrated terminal HexA residue. A glycopeptide derived from the N-terminal domain of perlecan encompassing the three previously known HS-sites (DDASGDGLGSGDVGSGDFQMVYFR) where found with three tetrasaccharide modifications (614.1694 Da), one methionine oxidation and one phosphate modification. The measured mass (4389.5071 Da) deviated +2.05 ppm from the theoretical value. Furthermore, the glycan was found modified with one phosphate group (PO_3_^−^) at a Xyl residue (peptide+Xyl+PO_3_, *m*/*z* 1340.5217; 2+) (Supplementary Fig. S3). The distinction between phosphate- (79.9663 amu) and sulfation modification (79.9568 amu) was feasible by examining the spectrum in a more narrow mass range. A mass shift of 212.0090 Da between *m*/*z* 1234.5172; 2+ and *m/z* 1340.5217; 2+ was observed in the range of *m*/*z* 1000 – 1400 (Supplementary Fig. S3). This demonstrates the presence of a xylose and a phosphate group (132.0423 + 79.9663 = 212.0086 Da), as opposed to a xylose and a sulfate group (132.0423 + 79.9568 = 211.9991 Da) (Supplementary Table S1). The automated Mascot search algorithm was constructed to identify if other HS-glycopeptides were modified with tetrasaccharides in the same sample preparation. The analysis was allowed to include tetrasaccharide structures ΔHexAGalGalXyl-O- without (614.1694 Da) or with one phosphate group attached (694.1358 Da). The search enabled the identification of HS-glycopeptides derived from mouse collagen XVIII, agrin and collagen XV, several which had the phosphate modification on the xylose residue (Supplementary Fig. S4) (Supplementary Table S1). Furthermore, the C-terminal HS-site of mouse perlecan was also found with a tetrasaccharide and further examination identified variants related to NH_3_-rearrangement (Supplementary Fig. S5). Similar NH_3_-rearrangements were also identified for the hexa-, octasaccharides, which eluted at ~46 min in Fig. 3. Taken together, the Mascot-assisted search analysis of the sample preparation enabled the identification of seven different mouse HS-glycopeptides derived from four different core proteins (perlecan, collagen XVIII, agrin and collagen XV).

### Identification of a hybrid proteoglycan site

**Figure 4:**
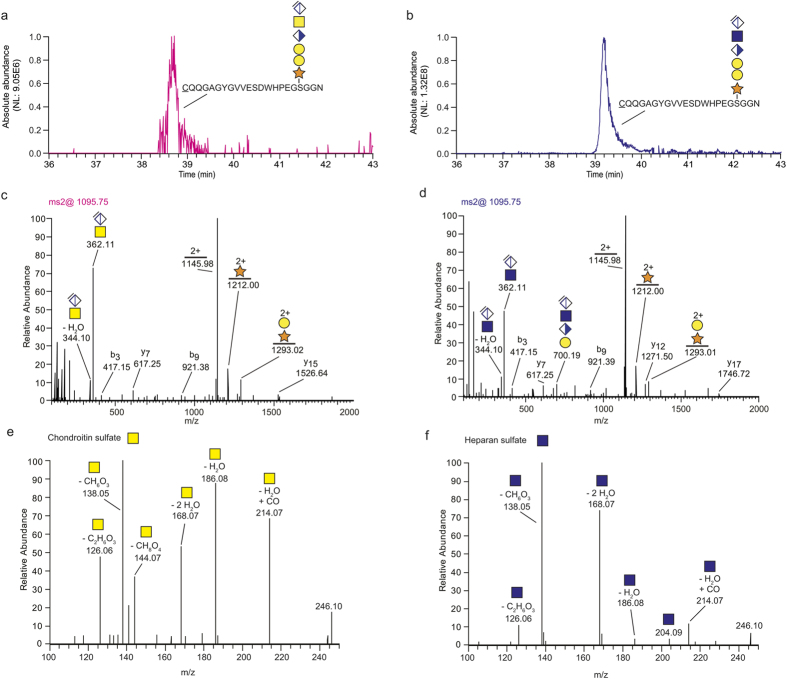
Identification of a hybrid proteoglycan site. (**a**,**b**) Extracted-ion current chromatograms of chondroitinase ABC- and heparinase digested perlecan samples revealed a C-terminal CS-glycopeptide (**a**) (*m/z* 1095.75; 3+) and a HS-glycopeptide (**b**) (*m/z* 1095.75; 3+), respectively, both eluting at ~39 min. (**c**,**d**) The MS2 spectra of the CS-glycopeptide (**c**) and HS-glycopeptide (**d**) provided almost identical peptide- and glycosidic fragmentation. (**e**,**f**) The oxonium-derived ion profile in the low mass range revealed higher intensities of *m/z* 126.06 and *m/z* 144.07 for the CS-glycopeptide (**e**) compared with the HS-glycopeptide showing higher intensities of *m/z* 138.05 and *m/z* 168.07 (**f**), thereby differentiating the presence of GalNAc- and GlcNAc-oxonium ions, respectively.

Since perlecan has been suggested to be a hybrid proteoglycan and as our initial SDS-PAGE analysis indicated the presence of CS, we wanted to determine which of the HS-sites may also be substituted with CS. GAG-glycopeptides were enriched by SAX-chromatography and eluted with high salt buffers as previously described. The collected fractions were divided in half and one part was digested with heparinase and the other with chondroitinase ABC. The samples were analyzed in consecutive order on the nLC-MS/MS. The general workflow for glycopeptide enrichment, the enzyme digestions, and the subsequent MS-analysis is illustrated in Supplementary Fig. S6. The chondroitinase-digested sample contained the anticipated hexasaccharide-substituted C-terminal glycopeptide (CQQGAGYGVVESDWHPEGSGGN), indicating the presence of a CS-substitution. The identified CS-glycopeptide eluted at ~39 min (Fig. 4a), similar to that of the C-terminal HS-glycopeptide of the heparinase digested sample (Fig. 4b), although of much lower intensity. The HCD-generated MS2-spectra of the CS- and HS-glycopeptides at *m/z* 1095.75; 3+ were virtually indistinguishable and both contained a prominent *m/z* 362.11 ion, as well as similar glycosidic and peptide fragments (Fig. 4c,d). However, GalNAc- and GlcNAc residues produce different oxonium ion profiles during HCD-fragmentation, which can be used for saccharide identification^25^. The GalNAc-derived oxonium fragments typically produce relatively higher intensities of *m/z* 126.06 and *m/z* 144.07 compared with GlcNAc-derived fragments. Oppositely, GlcNAc-derived oxonium fragments typically produce relatively higher intensities of *m/z* 138.05 and *m/z* 168.07. In accordance with this concept, the chondroitinase ABC digested sample revealed higher intensities of *m/z* 126.06 and *m/z* 144.07 compared with that of the heparinase digested sample, and vice versa for the m/z 138.05 and m/z 168.07 ions, thereby providing direct evidence for the structural identities of the two detected GAG-structures (CS and HS). Notably, the perlecan N-terminal HS-sites were not found to be substituted with CS, illustrated by the dominance of oxonium ion peaks at *m/z* 138.05 and 168.07 (Fig. 2b). Taken together, this suggests that the C-terminal site of mouse perlecan is indeed a ‘hybrid proteoglycan site’, carrying both HS and CS.

## Discussion

Despite great interest for HSPGs and CSPGs in biomedical research, there exists no effective method to experimentally determine their GAG attachment sites *in vivo*. Here we present a glycoproteomics approach that provides combined information of HS and CS linkage regions, and their attachment sites as well as the identities of the core protein. This method may facilitate studies on HS, CS and hybrid type PGs in various pathophysiological settings, as well as assist in elucidating the influence of a peptide code for GAG-biosynthesis.

Due to the complex nature of PGs, the GAG chains and the core protein are typically separated prior to structural analysis and although this procedure facilitates the analysis in some aspects, it precludes site-specific glycan information. SAX-chromatography is commonly used for enrichment for GAG chains as the positively charged matrix retains the anionic polysaccharide chains^26^,^27^. Similar to previous work, GAG-substituted glycopeptides rather than GAG chains or complete PGs were now enriched. Additionally, we have now built on our recently published methodology for CSPG characterization in order to specifically analyse HS modifications of strict HSPGs or hybrid type PGs^18^,^19^. Heparinase enzymes were used to reduce the length and structural complexity of the polysaccharides which results in the release of internal disaccharides and generation of a residual saccharide structure containing the linkage region of the HS chains still attached to the peptide. As heparinases act by elimination, they generate disaccharides containing Δ4,5 HexA and a Δ4,5 HexA at the non-reducing end of the residual saccharide structure^28^. Other approaches using heparinase for HS- and HSPG structural characterization has previously been described^29^,^30^. Some of these methods include composition analysis of the released disaccharides following heparinase digestion^31^. Such analysis provides a picture of the polysaccharides in terms of its constituent disaccharides^32^. However, as the disaccharides are derived from a mixture of HS chains of various core proteins this strategy does not provide site-specific compositional information. An antibody that reacts with neoepitopes of the heparinase-generated saccharide structures has also been described^30^. In combination with heparinase digestion, the antibody provides a general overview of expressed HSPGs in a given sample. However, no core protein identities are obtained using such antibody-based assay. The method described here has the advantage of providing site-specific glycan information together with core protein identities. This is all achieved in a single analysis and should thus be an important complement to previous methods.

The number of identified core proteins that carry HS chains is relatively low. Only seventeen proteins are known to carry HS, which is a very limited number compared to the number of proteins known to carry N- or O-linked glycosylations^33^,^34^,^35^. Although this difference may primarily reflect that the potential attachment sites in the proteome is far greater for N- and O-glycans compared with that of HS, a certain degree of research bias regarding detection methods may not be excluded. The introduction of glycoproteomics for site-specific analysis of N- and O-glycans has resulted in a significant increase in the number of identified glycoproteins^16^,^17^. We identified several HSPGs in a single sample preparation, illustrating that this method may potentially be routinely used to identify HSPGs in biologically relevant samples. An interactome analysis of agrin, collagen XV and collagen XVIII, identified in the perlecan-enriched sample now analyzed, demonstrates that these HSPGs interact with perlecan in a complex manner (Supplementary Fig. S7). Thus, as these components are integral parts of the basement membrane they are likely to be co-enriched with perlecan during the extraction procedure. Glycoproteomic analysis of CSPGs has enabled the identification of several novel CS core proteins, many of which were previously defined as prohormones^18^. Detailed analysis also revealed that the linkage region contained unexpected sialic acid and fucose modifications^19^. Whether further glycoproteomic analysis of HSPGs will result in the identification of novel core proteins, unforeseen linkage region complexity, or the discovery of novel functional classes of proteoglycans remains to be determined.

The term hybrid proteoglycans is used to denote core proteins which carry both HS and CS chains. However, it is often unclear on a peptide level whether a certain attachment site carries HS or CS chains, or both. The combined use of heparinase- and chondroitinase ABC, as used here, enabled the identification of a hybrid site only at the C-terminal end of mouse perlecan. MS2-fragmentation generated a prominent oxonium ion at *m***/***z* 362.11 for both glycopeptides, which corresponds to the terminal dehydrated disaccharide [ΔHexAHexNAc]^+^ of two possible hexasaccharide structures. The analysis of the HexNAc-derived oxonium ion profiles provided additional support to the saccharide identities (GlcNAc and GalNAc respectively). Such analysis has previously been used for N- and O-glycans and provides a direct evidence of the isomer identity, in contrast to the indirect evidence provided by the enzyme specificities^25^,^36^,^37^. This additional confirmation is important as the purity and specificities of the enzymes are not always optimal, and thus the possibility of cross-reactivity may not be excluded. Definite assignment of saccharide identities is therefore only feasible for hexasaccharide- or longer structures, as they generate unique finger-print oxonium ion patterns that serve as a direct proof of the isomer identities.

The identification of ‘hybrid sites’ is likely to be biologically important as HS and CS often display opposite effects on cell function^38^,^39^. In neurons, HS and CS control axonal generation through the interaction with a protein tyrosine phosphatase, and while HS has been shown to promote neurogenesis CS has an inhibitory effect^39^. One may speculate whether the separate C-terminal HS and CS polysaccharides on perlecan, as shown here, induce opposite effects on cell function. Apart from its structural role in basement membranes, perlecan also contributes to the regulation of blood vessel growth^21^. The proteolytic processing of its C-terminal domain liberates endorepellin, a bioactive domain with angiostatic activity^40^. The activity has been assigned to the laminin-like globular (LG3) domain, which is generated upon cleavage of endorepellin by bone morphogenic protein 1 (BMP1)^41^. Interestingly, BMP1 cleaves endorepellin at a position in close proximity (+3 residues) to the hybrid GAG-site. One may speculate whether the GAG-site influences the regulation of BMP1 cleavage of perlecan, and if so, whether CS and HS chains may have opposing roles in such a process.

Interestingly, the fixed HS-sites of the N-terminal domain (DDASGDGLGSGDVGSGDFQMVYFR) display a different peptide motif compared to the hybrid site in the C-terminal domain of perlecan (CQQGAGYGVVESDWHPEGSGGN). Although no straightforward consensus motifs exist to predict potential GAG-sites, certain features have been identified to influence the selection of HS-synthesis and CS-synthesis, respectively. HS-sites typically contain repetitive SG-motifs and sometimes also a tryptophan residue in the vicinity of the SG-repeats^42^. In contrast, CS-sites typically contain only a single SG-attachment site with a cluster of acidic residues in close proximity^10^. Whereas the fixed HS sites in the N-terminal end of perlecan largely conform to this notion, the hybrid site in the C-terminal end does not conform to neither of the classical HS nor the established CS-promoting sequences. Although the CQQGAGYGVVESDWHPEGSGGN sequence has an apparent resemblance of a CS-promoting sequence due to its single SG-dipeptide, the peptide has relatively few acidic residues and contains a tryptophan residue in the vicinity to the attachment site, which are features more expected for fixed HS-sites. Future studies will reveal if this motif is present in other core proteins, and if so, whether they also code for hybrid GAG-sites. However, the amino acid sequence alone does not completely differentiate the GAG-selection process, as some sites are unoccupied and the determination of HS vs. CS-glycosylation appears only partly encoded by the peptide sequence. Serglycin, the major proteoglycan of mast cells, contains multiple clusters of SG-SG dipeptides and may carry both heparin (a highly sulfated variant of HS) and CS^43^. Mast cells derived from the peritoneum contain only heparin whereas mast cells of the lung contain both heparin and CS, suggesting that the core protein substitution varies with the differentiation and functional status of the cells^44^. It is possible that other post-translational modifications, such as N-glycans and mucin-type O-glycans, in vicinity to the GAG-site may also influence the biosynthetic process. We have previously identified combined site-specific characterization of CS linkage regions and mucin-type O-glycans on the very same glycopeptide of bikunin (protein AMBP)^19^. The basis of a peptide code and the influence of post-translational modifications adjacent to the attachment site is something that can be tackled afresh with our new methodological approach.

A long-asked question is whether the core protein sequence influences the final structure of the synthesized HS chains. For instance, does the HS chains of the N-terminal end of perlecan display different fine-structures compared with the HS chain of the hybrid site on the C-terminal end? Interestingly, our results show that site-specific sequencing of longer structures is also feasible with our method, including analysis of octasaccharides and decasaccharides (Fig. 3). Notably, no sulfate- or phosphate modifications were identified on these structures, indicating that the first proportion of the HS chain on the C-terminal site may be relatively non-modified. Our finding is in keeping with previous studies, which demonstrates that the proximal region of HS close to the protein core may sometimes be extended with non-sulfated domains of about 10 disaccharides in length^45^,^46^. Moreover, with the use of shorter heparinase incubation time, sequencing of longer structures is likely to be feasible. Although site-specific detailed sequencing of full-length structures will be very challenging, the analysis of, perhaps, the first 14–16 residues of the HS chains should be feasible and would provide insights into whether the attachment site peptide sequence influences the fine-structure of the polysaccharides. The idea of site-specific HS polysaccharide structures relates to the concept of specificity for HS-protein interactions and is currently a debated area^47^.

As HS chains influence the spatiotemporal organization of various physiological processes, this implicates a degree of specificity between the polysaccharides and the interacting ligands. Indeed, evidence shows that some activities clearly need a distinct sulfation pattern, whereas other interactions display lower degree of specificity and requires less stringent sulfate distribution^3^. If any given core protein or peptide sequence is associated with a unique fine-structure this information will likely contribute to our understanding of the concept of specificity and provide a novel theoretical framework for the understanding of HS-protein interactions. We suggest that the method presented herein open new possibilities for site-specific characterization of HS, CS and hybrid proteoglycans.

In this work we present a novel glycoproteomic approach that provides site-specific identification and differentiation of HS- and CS-sites *in vivo*. The use of heparinase and chondroitinase ABC enabled the identification and differentiation between consensus HS sites and hybrid GAG sites, and inspection of the amino acid sequence indicated that certain peptide motifs may encode for the chain type selection process. The method may be used to address fundamental questions in basic biology, as well as assist in elucidating the functional roles of peptide sequences in the biosynthesis of proteoglycans.

## Methods

### Perlecan GAG-peptide preparation

Twenty microgram of the commercial perlecan sample, isolated from Engelbreth-Holm-Swarm mouse sarcoma, (H4777, Sigma-Aldrich) was trypsinized using an in-solution digestion protocol. Briefly, the sample was incubated for 10 min with Protease Max surfactant trypsin enhancer (0.02 % final concentration) (Promega) in 50 mM NH_4_HCO_3._ The sample was thereafter reduced with DTT (5 mM) and alkylated with iodoacetamide (15 mM). Additional Protease Max surfactant was then added (0.03 % final concentration) and the sample was trypsinized over night (37 °C) with 20 μg trypsin (Promega). The trypsin-digested sample was enriched for GAG-peptides using SAX-chromatography (Vivapure, Q Mini H), as described previously^19^. Briefly, the sample was diluted in 10 mL coupling buffer (50 mM NaAc, 200 mM NaCl, pH 4.0) and applied onto the column and spun at 1000× g for 2 min. The procedure was repeated until all sample volume had been applied onto the column. The column was washed with 400 μL of a low-salt wash solution (50 mM Tris-HCl, 200 mM NaCl, pH 8.0) and the GAG-peptides were eluted step-wise with three buffers of increasing NaCl-concentrations and pH; (1) 50 mM NaAc, 400 mM NaCl, pH 4.0, (2) 50 mM Tris-HCl, 800 mM NaCl, pH 8.0 and (3) 50 mM Tris-HCl, 1600 mM NaCl, pH 8.0. The collected fractions were desalted using a PD10-column (GE Healthcare) and individually subjected to heparinase or chondroitinase ABC degradation or without glycosidase treatment. For the heparinase digestion, 0.3 mU each of heparinase I (H2519, Sigma Aldrich) and heparinase III (H8891, Sigma Aldrich) were used together. The samples were incubated for 24 hrs. at 37 °C in 40 μL digestion buffer (50 mM NaAc, pH 7.0, 0.1 mM CaCl_2_). For the chondroitinase ABC digestion, 0.3 mU of chondroitinase ABC (C3667, Sigma-Aldrich) was used and incubated for 24 hrs. at 37 °C in 40 μL digestion buffer (55 mM NaAc, pH 8.0). The actions of heparinase (I and III), chondroitinase ABC and trypsin were monitored with SDS-PAGE. Three micrograms of perlecan incubated with and without trypsin was used as control. The samples were mixed with 5xSDS sample buffer and loaded onto a 4–20% Novex Tris-Glycine gel (Invitrogen, Carlsbad, CA). After electrophoresis separation at 100 V for 1 h the gel was stained with Alcian blue and thereafter scanned.

### LC-MS/MS analysis with Q Exactive and Orbitrap Fusion Tribrid

Prior to MS-analysis, the samples were desalted again using a C18 spin column (8 mg resin, Pierce). The samples were analyzed on a Q Exactive (QE) mass spectrometer (MS) coupled to an Easy-nLC 1000 (Thermo Fisher Scientific, Inc., Waltham, MA, USA) and an Orbitrap Fusion Tribrid mass spectrometer interfaced to an Easy nanoLC1000 (Thermo Fisher Scientific). Glycopeptides were separated using an in-house constructed pre-column and analytical column set up (45 mm × 0.100 mm I.D. and 250 mm × 0.075 mm I.D., respectively) packed with 1.7 or 3 μm Reprosil-Pur C18-AQ particles (Dr. Maisch GmbH, Ammerbuch, Germany). An acetonitrile (ACN) gradient was run at 200 nL/min from 7 to 80 % B-solvent (ACN in 0.2% formic acid) over 70 or 90 min, with A-solvent of 0.2 % formic acid. Ions were generated and injected into the MS instrument under a spray voltage of 1.6 kV in positive ion mode. MS scans were performed at 70 000 resolution (QE), 120 000 resolution (Fusion), (at *m/z* 200), with a mass range of *m/z* 600–2000 (QE) or *m/z* 400–1500 (Fusion). MS/MS analysis was performed in a data-dependent mode, with the top 5 (QE) or top speed 3s (Fusion) of the most abundant doubly or multiply charged precursor ions in each MS scan selected for fragmentation (MS2) by high energy collision dissociation (HCD) of a normalized collision energy (NCE) value at levels of 20, 25, 30 and 35%. For MS2 scans an isolation window of 2–2.5 Da was used and the resolution of detection of fragment ions was 35000 (QE) and 30000 (Fusion), respectively.

### Mascot search for GAG-glycopeptides

The MS data were processed using Mascot Distiller and searches for GAG-glycopeptides were performed as previously described^19^. Briefly, the HCD.raw spectra files were converted to Mascot.mgf format using Mascot distiller (version 2.3.2.0, Matrix Science, London, UK). The ions were presented as singly protonated in the output Mascot file. Searches were performed using an in-house Mascot server (version 2.3.02) with the enzyme specificity set to *trypsin* and then to “semitrypsin”, meaning that the program will search for peptides that display tryptic specificity at one terminus, whereas the other terminus may be a non-tryptic cleavage site. The searches were performed on human sequences of the UniprotKB (87,613, sequences, 13/3/2013). The instrument parameter was set to consider the MH+ form of b- and y-ions and their losses of NH_3_ and H_2_O. The peptide tolerance was set to 10 parts per million (ppm) and fragment tolerance was set to 0.01 Da. The searches were allowed to include variable modifications at serine residues of the residual hexasaccharide structure [HexA(-H_2_O)GlcNAcGlcAGalGalXyl-O-] with 0 (C_37_H_55_NO_30_, 993.2809 Da), 1 (C_37_H_55_NO_33_S, 1073.2377 Da), or 2 (C_37_H_55_NO_36_S_2_, 1153.1945 Da) sulfate groups attached. Additionally, searches were allowed to include variable modifications at serine residues of a residual tetrasaccharide structure [HexA(-H_2_O)GalGalXyl-O-] with 0 (C_23_H_34_O_19_, 614.169429 Da) or 1 phosphate group attached (C_23_H_35_O_22_P, 694.135760 Da). Moreover, ‘neutral losses’ of the same masses were implemented to the search constraints, as O-glycan modification is absent in the obtained peptide fragments at a NCE of 30%.

### Data evaluation

All generated GAG-glycopeptide hits were manually evaluated according to previous established criteria for CS-glycopeptides^19^. HS-glycopeptide hits were evaluated using following criteria: (1) The presence of HexNAc-generated oxonium ions, specifically *m***/***z* 362.11. (2) At a NCE of 20% the MS2-spectra should also display stepwise glycosidic fragmentation of the linkage region and/or the peak(s) corresponding to the deglycosylated peptide ions. The accuracy of the precursor mass should not deviate more than 5 ppm from the theoretical mass value.

## Additional Information

**How to cite this article**: Noborn, F. *et al*. Site-specific identification of heparan and chondroitin sulfate glycosaminoglycans in hybrid proteoglycans. *Sci. Rep*. **6**, 34537; doi: 10.1038/srep34537 (2016).

## Supplementary Material

Supplementary Information
