# Supplementary material for: Site-specific identification of heparan and chondroitin sulfate glycosaminoglycans in hybrid proteoglycans
Source: Sci Rep. 2016 Oct 3;6:34537. doi: 10.1038/srep34537 (PMC5046109; doi:10.1038/srep34537)
Supplement: Supplementary Information [file srep34537-s1.pdf]

Supplementary Information

Site-specific identification of heparan and chondroitin sulfate glycosaminoglycans in hybrid proteoglycans

Fredrik Noborn[1], Alejandro Gomez Toledo[1], Anders Green[1], Waqas Nasir[1], Carina Sihlbom[2], Jonas Nilsson[1], and Göran Larson[1,*]

[1]Department of Clinical Chemistry and Transfusion Medicine, Institute of Biomedicine, University of Gothenburg, Sahlgrenska University Hospital, SE-413 45 Gothenburg, Sweden.

[2]Proteomics Core Facility, Sahlgrenska Academy, University of Gothenburg, Box 413, SE-405 30, Sweden.

*To whom correspondence should be addressed: Göran Larson, Department of Clinical Chemistry and Transfusion Medicine, Institute of Biomedicine, University of Gothenburg, Bruna Stråket 16, Sahlgrenska University Hospital, SE-413 45 Gothenburg, Sweden. Tel: +46-(0)31 342 1330; Fax: +46-(0)31-82 84 58; E-mail: goran.larson@clinchem.gu.se

Running title: Identification of glycosaminoglycan attachment sites

Agrin (Mus musculus) (A2ASQ1)
MS2 @ 1312.80 (3+)

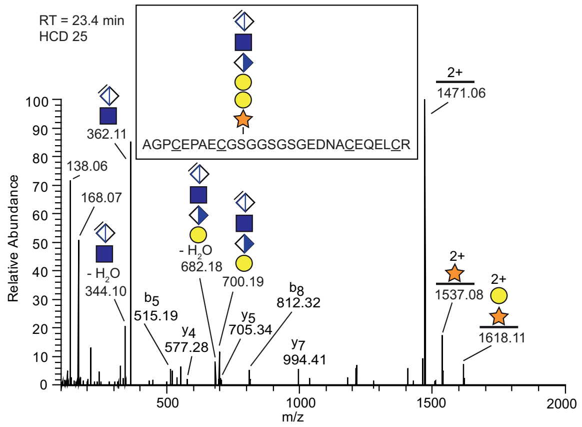

Collagen alpha-1(XVIII) chain (Mus musculus) (P39061)
MS2 @ 1131.39 (3+)

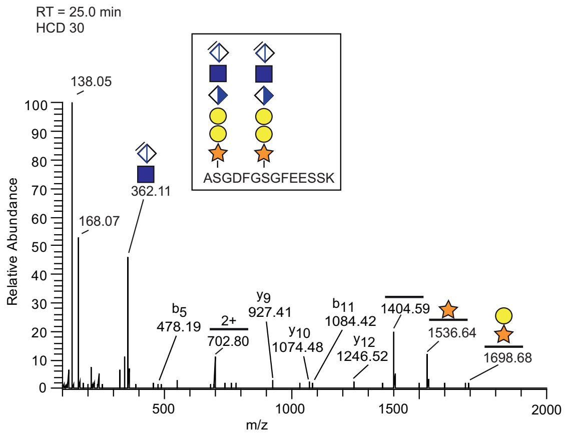

Collagen alpha-1(XVIII) chain (Mus musculus) (P39061)
MS2 @ 1029.09 (3+)

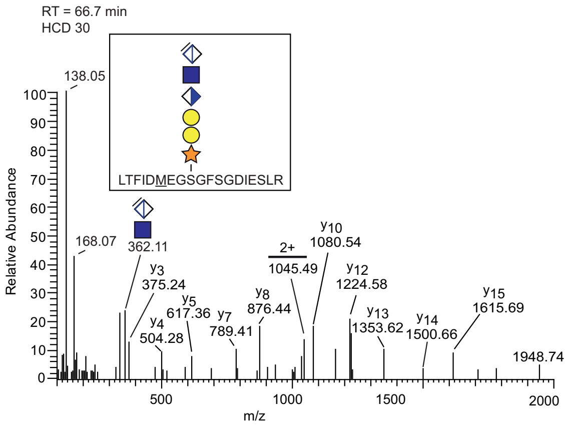

Collagen alpha-1(XVIII) chain (Mus musculus) (P39061)
MS2 @ 1128.78 (3+)

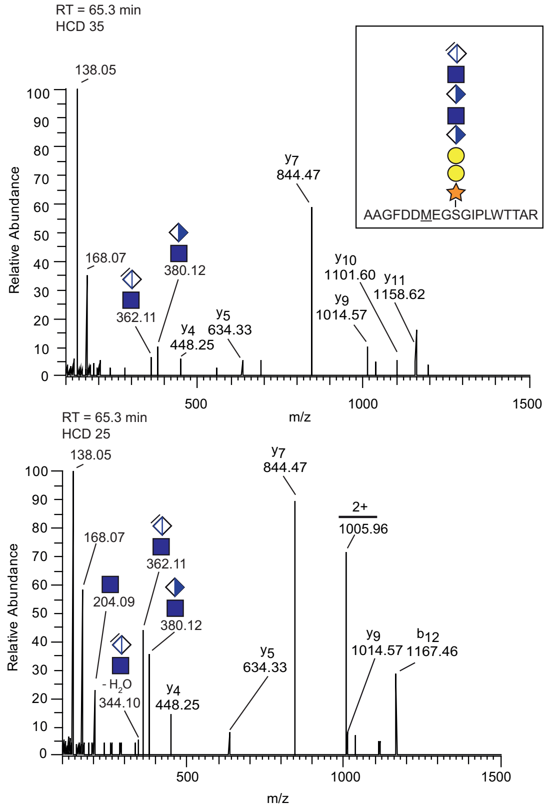

Figure S1. Fragment mass spectra of the identified HS-glycopeptides. A perlecan sample derived from Engelbreth-Holm-Swarm mouse sarcoma was analyzed for the presence of HSPGs. The sample was digested with trypsin and enriched for GAG-glycopeptides using strong anion exchange (SAX) chromatography. The GAG-glycopeptides were eluted from the column using three buffers of increasing sodium chloride concentration (0.4 M NaCl, 0.8 M NaCl and 1.6 M NaCl). The resulting fractions were digested with a mixture of heparinases and analyzed with nLC-MS/MS in a 90 min program. A typical HCD spectrum of each identified HS-glycopeptide is shown. The retention time (RT) and the fragmentation energy level for each precursor ion are indicated (NCE 25, 30 or 35%). The lengths of the identified structures were either hexasaccharides or octasaccharides. All the HS-glycopeptides were identified in the 0.8 M fraction.

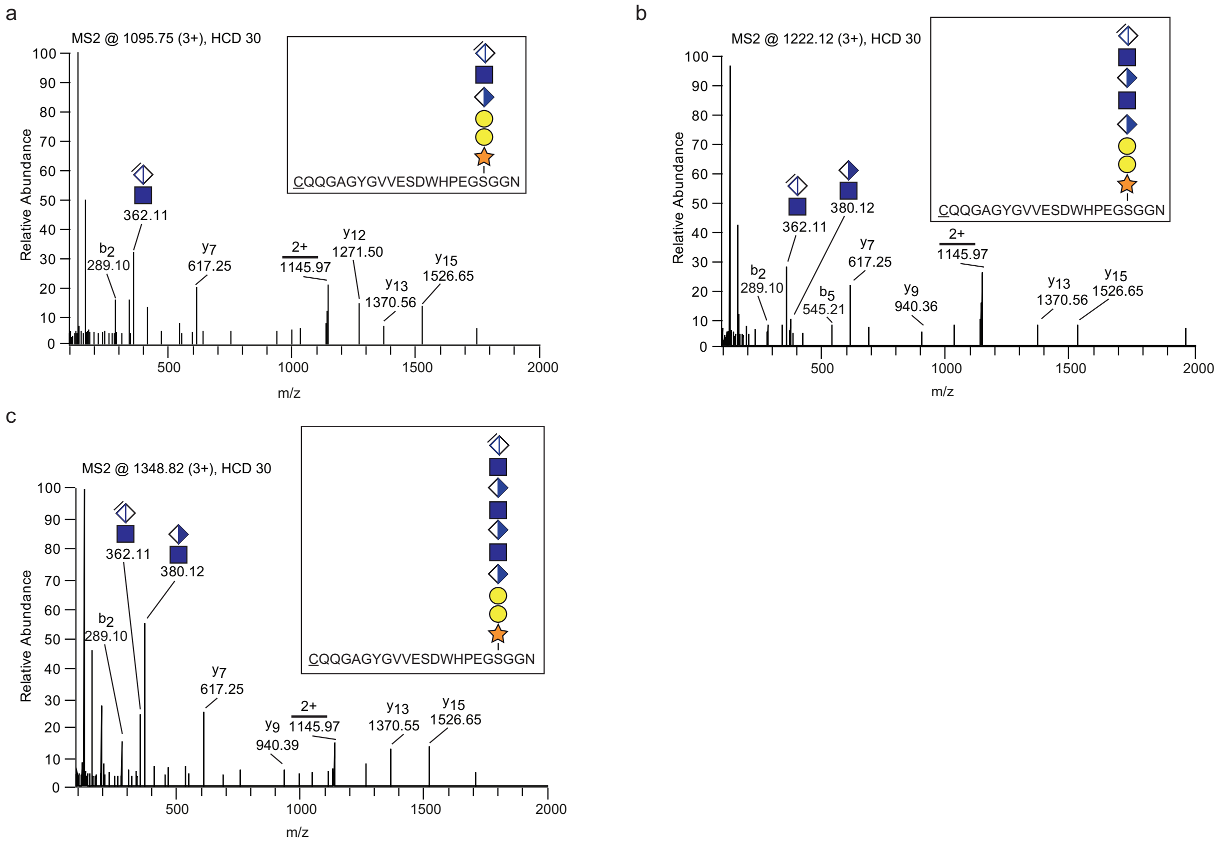

Figure S2. Identification of a HS-site in the C-terminal end of perlecan. nLC-MS/MS analysis of a heparinase-digested perlecan sample revealed a HS-site in the C-terminal end of the protein. A precursor ion of *m/z* 1095.75; 3+ was identified which corresponds to the peptide sequence of (CQQGAGYGVVESDWHPEGSGGN) and a residual HS-hexasaccharide structure generated by the heparinase digestion. Additional precursor ions with the saccharide structures of octasaccharides (*m/z* 1222.12; 3+) and decasaccharides (*m/z* 1348.82; 3+) were also identified. The MS fragment spectra of these extended structures displayed glycosidic- and peptide fragmentation similar to that of the hexasaccharide, including the diagnostic 362-ion. In addition, a prominent *m/z* 380.12 ion was observed, which corresponds to internal disaccharide fragmentation of the HS-chain $[GlcAGlcNAc]^{+}$. Notably, no precursor ions with the additional mass of a sulfate modification were observed.

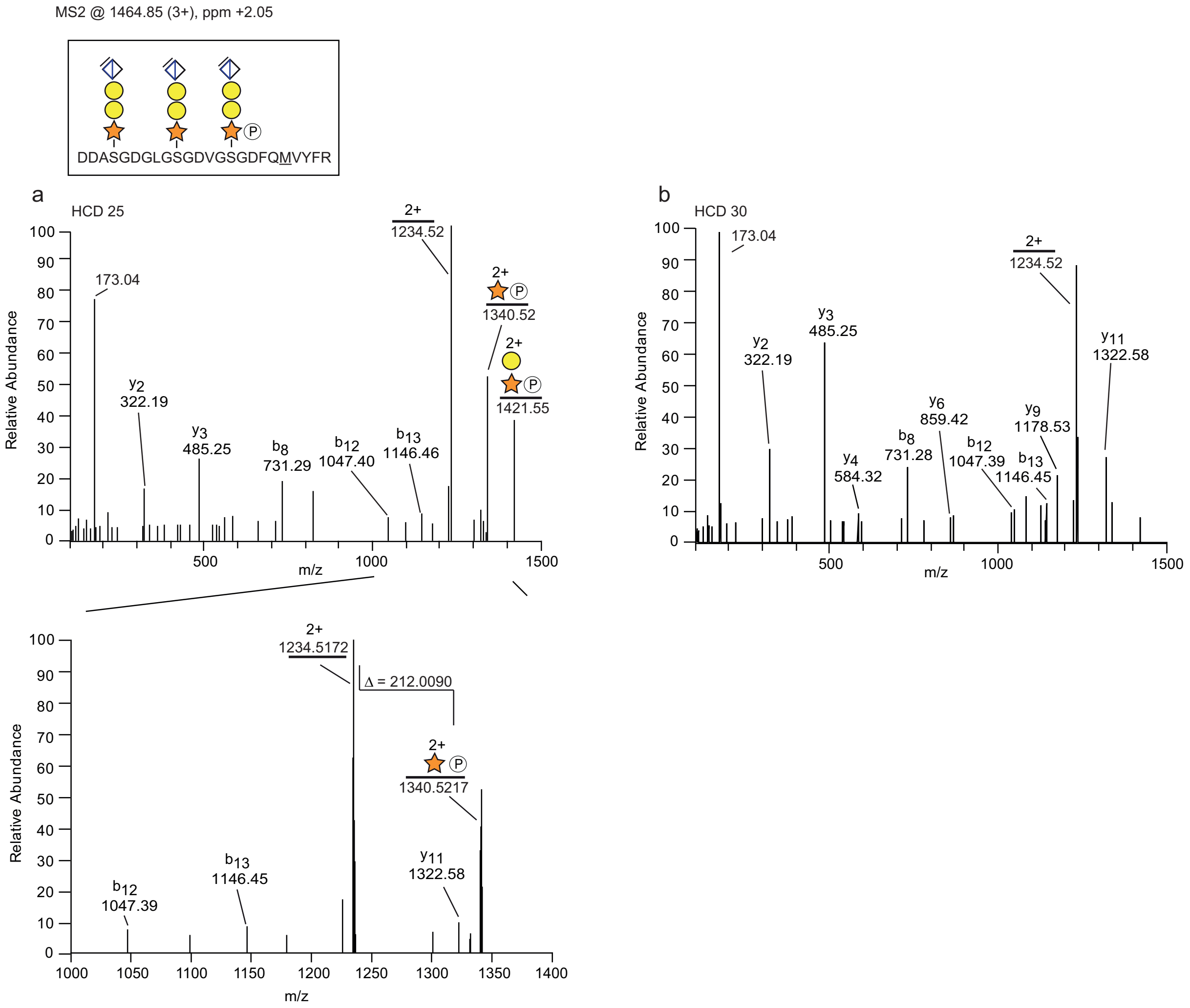

Figure S3. Identification of HS-tetrasaccharide structures. The heparinase-digestion of HS-glycopeptides also generated residual tetrasaccharide structures that were composed of the linkage region with a dehydrated terminal HexA residue. (a-b) A glycopeptide derived from the N-terminal domain of perlecan encompassing the three previously known HS-sites (DDASGDGLGSGDVGSGDFQMVYFR) where found with three tetrasaccharide modifications (614.169429 Da), one methionine oxidation and one phosphate modification. HCD spectra of the identified HS-glycopeptide are shown at a fragmentation energy level of NCE 25 % (a) and 30 % (b). The distinction between phosphate- and sulfation modification was feasible by examining the spectrum in a more narrow mass range. A mass shift of 212.0090 Da between *m/z* 1234.5172; 2+ and *m/z* 1340.5217; 2+ was observed in the range of *m/z* 1000 – 1400 (a, enlarged view). This demonstrates the presence of a xylose and a phosphate group (132.0423 + 79.9663 = 212.0086 Da), as

opposed to a xylose and a sulfate group (132.0423 + 79.9568 = 211.9991 Da). The finding that the xylose residue is modified with a phosphate group is in keeping with the literature, as structural studies of both CS- and HS proteoglycans have reported phosphate modifications on xylose residues[1, 2]. Moreover, a kinase (FAM20B) and a phosphatase (XYLP) have been identified that are involved in the specific addition and removal of phosphate groups on xylose residues of the linkage region [3, 4].

Agrin (Mus musculus) (A2ASQ1)
MS2 @ 1391.16 (3+)

HCD 30

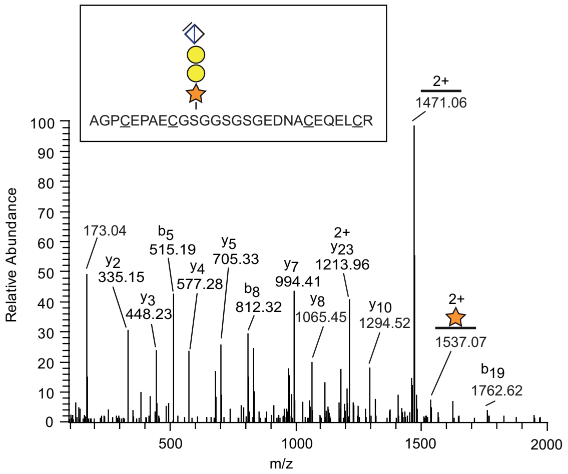

Collagen alpha-1(XVIII) chain (Mus musculus) (P39061)
MS2 @ 1134.44 (3+)

HCD 25

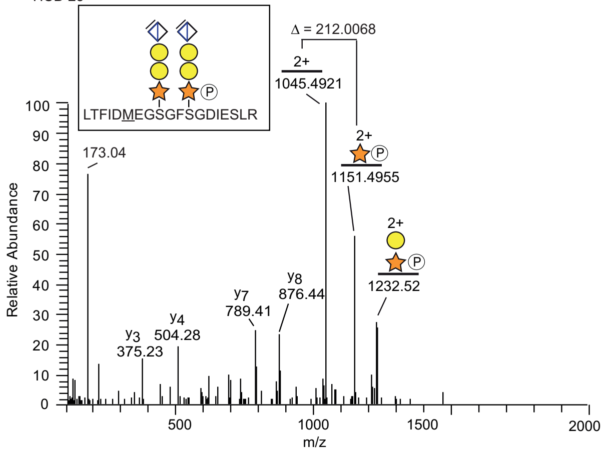

Collagen alpha-1(XVIII) chain (Mus musculus) (P39061)
MS2 @ 1317.47 (2+)

HCD 30

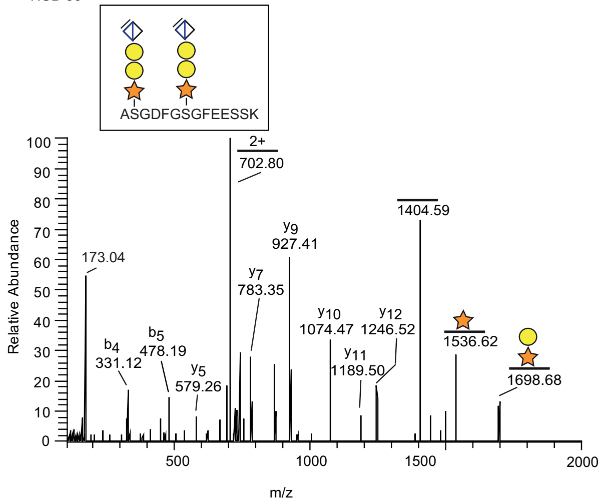

Collagen alpha-1(XVIII) chain (Mus musculus) (P39061)
MS2 @ 1211.96 (3+)

HCD 25

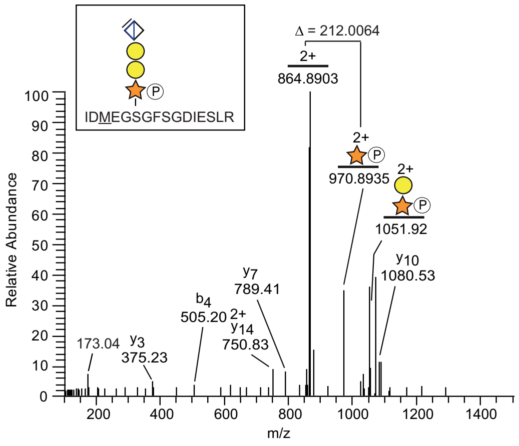

Collagen alpha-1 (XV) (Mus musculus) (O35206)
MS2 @ 930.35 (2+)

HCD 25

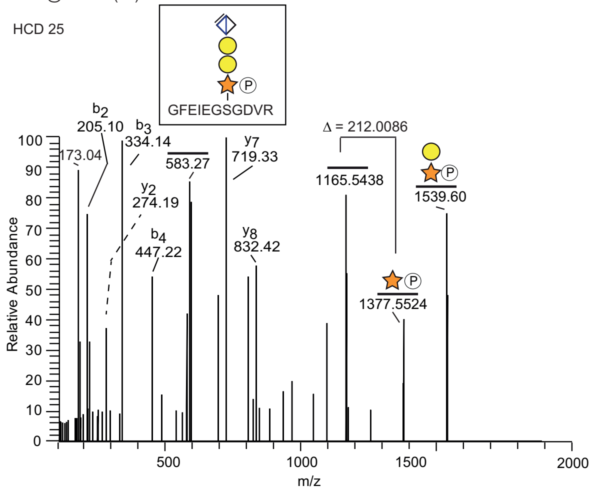

Figure S4. Fragment mass spectra of the identified HS-tetrasaccharide glycopeptides. A typical HCD spectrum of each identified HS-glycopeptide is shown. The fragmentation energy level for each precursor ion is indicated (NCE 25, 30 or 35%).

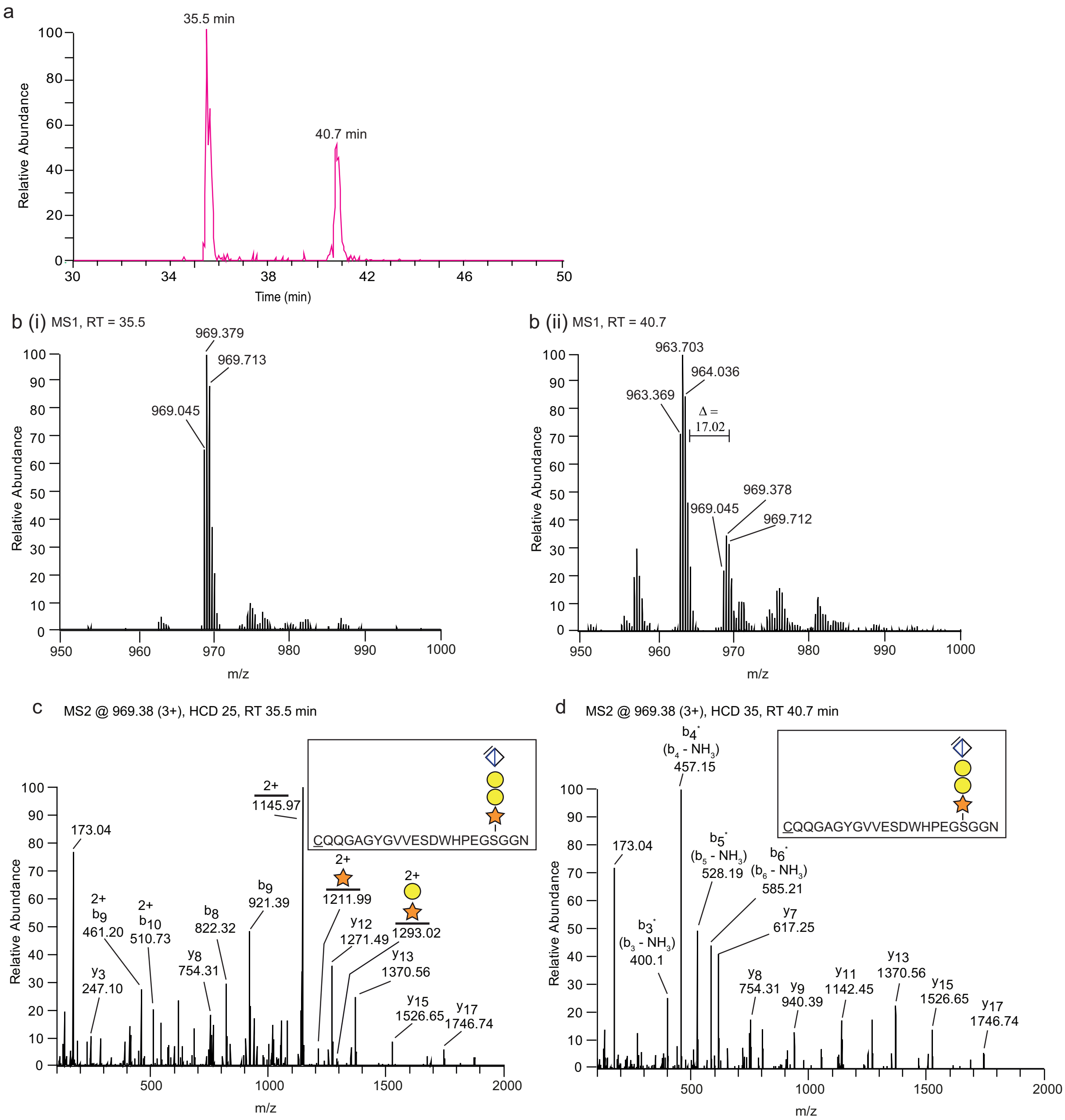

Figure S5. Identification of a HS-tetrasaccharide structure in the C-terminal end of perlecan. (a) An extracted-ion current chromatogram of a heparinase-digested perlecan sample revealed a HS-tetrasaccharide structure (*m/z* 969.04; 3+) at the C-terminal perlecan site, eluting as two distinct peaks at RT = 35.5 min and 40.7 min. (b) Detailed inspection of the precursor ions indicated that the late eluting peak (b, ii) relates to an NH_3-adduct (delta mass = + 17.02 Da) of a HS-tetrasaccharide structure with a mass of 17.02 Da less (*m/z* 963.37; 3+) than the original structure at *m/z* 969.04; 3+. Peptides containing N-terminal cysteine residues are prone to undergo cyclization of

carboxamidomethylated cysteine residues, resulting in the loss of an ammonium group (17.02 Da)[5]. As the peptide sequence of the C-terminal HS-site of perlecan has a cysteine residue in the N-terminus (CQQGAGYGVVESDWHPEGSGGN), the late eluting peak is therefore likely the result of an N-terminal loss of a NH_3-group with the subsequent adduct formation of a NH_3-group to the glycopeptide. This NH_3-rearrangement gives the glycopeptide a different chromatographic behavior and proposes an explanation to the two distinct chromatographic peaks observed. (c-d) Fragment mass spectra of the precursor ions of the early- and late eluting peaks. (c) The precursor ion of the early eluting peak (RT = 35.5 min) display a fragmentation pattern where several b- and y-ions are observed. (d) In contrast, the precursor ion of the late eluting peak (RT = 40.7 min) display only b*-ions and y-ions. The b*-ions are the result of the loss of an N-terminal NH_3-group, which is consistent with the proposed cyclization of a carboxamidomethylated cysteine residue and the subsequent loss of an ammonia group.

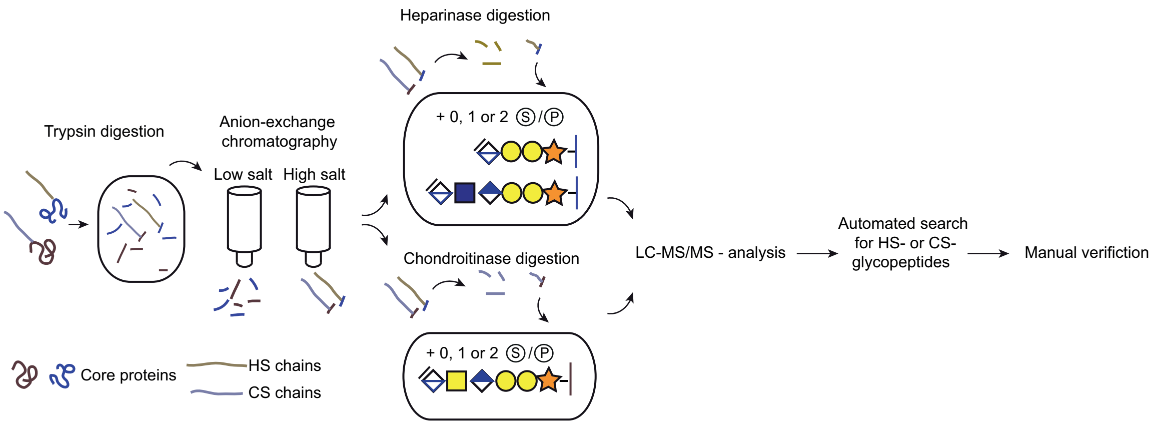

Figure S6. Work flow for enrichment, analysis and identification of HSPGs and CSPGs. General workflow from sample preparation to nLC-MS/MS-analysis and the interpretation of spectra.

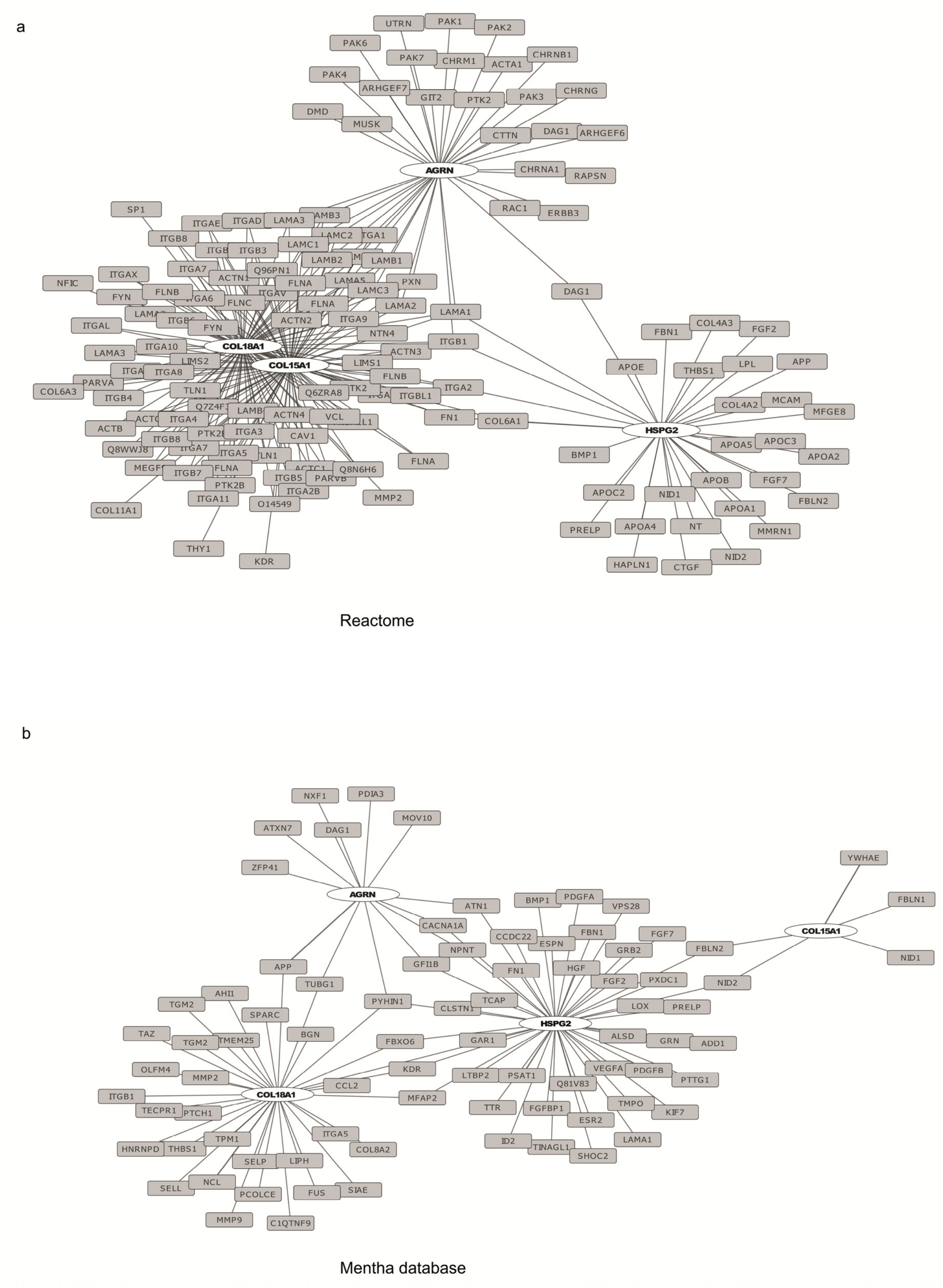

Figure S7. Network interactome analyses of the identified heparan sulfate proteoglycans. The identified proteoglycans (Perlecan, HSPG2; Agrin, AGRN; Collagen XIII, COL18A1) are essential for

the assembly and maintenance of the extracellular matrix. They are predicted to act on similar pathways as illustrated by their interconnections in the pathway Reactome Database (a) and have also been shown to physically interact with each other, directly or indirectly, as illustrated by their connections in the physical interaction database Mentha (b).

Supplementary Table S1
The deviation of identified mass shifts (xylose + PO_3H) on HSPGs

Identified structures (experimental mass) (m/z; charge)	Spectra	Theoretical delta mass[a] (Da)	Experimental delta mass[b] (Da)	Experimental delta mass error[c] (Da)	Experimental relative delta mass error[d] (ppm)	Theoretical delta mass[e] (non-identified modification) (Da)	Theoretical delta mass error[f] (non-identified modification) (Da)	Theoretical relative delta mass error[g] (non-identified modification) (ppm)
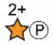 + peptide (1340.5217; 2+)	Sup. Fig. S3, Perlecan	212.0086 (Xyl + PO_3H)	212.0090	+ 0.0004	+ 1.9	211.9991 (Xyl + SO_3)	+ 0.0099	+ 46.7
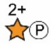 + peptide (1151.4955; 2+)	Sup. Fig. S4, Collagen alpha-1 (XVIII) chain	212.0086 (Xyl + PO_3H)	212.0068	- 0.0018	- 8.5	211.9991 (Xyl + SO_3)	+ 0.0077	+ 36.2
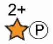 + peptide (970.8935; 2+)	Sup. Fig. S4, Collagen alpha-1 (XVIII) chain	212.0086 (Xyl + PO_3H)	212.0064	- 0.0022	- 10.4	211.9991 (Xyl + SO_3)	+ 0.0073	+ 34.4
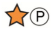 + peptide (1377.5524; 1+)	Sup. Fig. S4, Collagen alpha-1 (XV) chain	212.0086 (Xyl + PO_3H)	212.0085	- 0.0001	- 0.47	211.9991 (Xyl + SO_3)	+ 0.0094	+ 44.3

[a]Theoretical delta mass for loss of a xylose with a phosphate modification.

[b]Experimentally observed delta mass for the proposed loss of xylose with a phosphate modification.

[c]The experimental delta mass error (Da) denotes the difference between the experimental- and theoretical delta masses using monoisotopic ions.

[d]The experimental relative delta mass error (ppm) denotes the difference between the experimental- and theoretical delta mass error relative to the theoretical mass.

[e]The theoretical delta mass was calculated for a xylose with a sulfate modification.

[f] The theoretical delta mass error (Da) denotes the difference between the experimental- and theoretical delta mass, calculated for a theoretical sulfate modification.

[g]The theoretical relative delta mass error (ppm) denotes the difference between the experimental- and theoretical delta mass error relative to the calculated theoretical mass of a xylose with a sulfate modification.

References

1. Oegema, T.R., Jr., Kraft, E.L., Jourdian, G.W. & Van Valen, T.R. Phosphorylation of chondroitin sulfate in proteoglycans from the swarm rat chondrosarcoma. *J Biol Chem* **259**, 1720-1726 (1984).

2. Fransson, L.A., Silverberg, I. & Carlstedt, I. Structure of the heparan sulfate-protein linkage region. Demonstration of the sequence galactosyl-galactosyl-xylose-2-phosphate. *J Biol Chem* **260**, 14722-14726 (1985).

3. Koike, T., Izumikawa, T., Tamura, J. & Kitagawa, H. FAM20B is a kinase that phosphorylates xylose in the glycosaminoglycan-protein linkage region. *Biochem J* **421**, 157-162 (2009).

4. Koike, T., Izumikawa, T., Sato, B. & Kitagawa, H. Identification of phosphatase that dephosphorylates xylose in the glycosaminoglycan-protein linkage region of proteoglycans. *J Biol Chem* **289**, 6695-6708 (2014).

5. Krokhin, O.V., Ens, W. & Standing, K.G. Characterizing degradation products of peptides containing N-terminal Cys residues by (off-line high-performance liquid chromatography)/matrix-assisted laser desorption/ionization quadrupole time-of-flight measurements. *Rapid Commun Mass Spectrom* **17**, 2528-2534 (2003).
